# Supplementary material for: Resistance of Transgenic Maize Cultivars to Mycotoxin Production—Systematic Review and Meta-Analysis
Source: Toxins (Basel). 2024 Aug 22;16(8):373. doi: 10.3390/toxins16080373 (PMC11359299; doi:10.3390/toxins16080373)

SUPPLEMENTARY MATERIAL

Table S1. PRISMA checklist

Section and Topic	Item #	Checklist item	Page where item is reported
**TITLE**			
Title	1	Identify the report as a systematic review.	1
**ABSTRACT**			
Abstract	2	See the PRISMA 2020 for Abstracts checklist.	1
**INTRODUCTION**			
Rationale	3	Describe the rationale for the review in the context of existing knowledge.	2
Objectives	4	Provide an explicit statement of the objective(s) or question(s) the review addresses.	3
**METHODS**			
Eligibility criteria	5	Specify the inclusion and exclusion criteria for the review and how studies were grouped for the syntheses.	3 - 4
Information sources	6	Specify all databases, registers, websites, organisations, reference lists and other sources searched or consulted to identify studies. Specify the date when each source was last searched or consulted.	4
Search strategy	7	Present the full search strategies for all databases, registers and websites, including any filters and limits used.	4
Selection process	8	Specify the methods used to decide whether a study met the inclusion criteria of the review, including how many reviewers screened each record and each report retrieved, whether they worked independently, and if applicable, details of automation tools used in the process.	4
Data collection process	9	Specify the methods used to collect data from reports, including how many reviewers collected data from each report, whether they worked independently, any processes for obtaining or confirming data from study investigators, and if applicable, details of automation tools used in the process.	4
Data items	10a	List and define all outcomes for which data were sought. Specify whether all results that were compatible with each outcome domain in each study were sought (e.g. for all measures, time points, analyses), and if not, the methods used to decide which results to collect.	4
	10b	List and define all other variables for which data were sought (e.g. participant and intervention characteristics, funding sources). Describe any assumptions made about any missing or unclear information.	4
Study risk of bias	11	Specify the methods used to assess risk of bias in the included studies, including details of the tool(s) used, how many	4 – 5

Section and Topic	Item #	Checklist item	Page where item is reported
assessment		reviewers assessed each study and whether they worked independently, and if applicable, details of automation tools used in the process.	
Effect measures	12	Specify for each outcome the effect measure(s) (e.g. risk ratio, mean difference) used in the synthesis or presentation of results.	4 – 5
Synthesis methods	13a	Describe the processes used to decide which studies were eligible for each synthesis (e.g. tabulating the study intervention characteristics and comparing against the planned groups for each synthesis (item #5)).	5
	13b	Describe any methods required to prepare the data for presentation or synthesis, such as handling of missing summary statistics, or data conversions.	5 – 6
	13c	Describe any methods used to tabulate or visually display results of individual studies and syntheses.	3 - 5
	13d	Describe any methods used to synthesize results and provide a rationale for the choice(s). If meta-analysis was performed, describe the model(s), method(s) to identify the presence and extent of statistical heterogeneity, and software package(s) used.	4 - 8
	13e	Describe any methods used to explore possible causes of heterogeneity among study results (e.g. subgroup analysis, meta-regression).	8
	13f	Describe any sensitivity analyses conducted to assess robustness of the synthesized results.	5
Reporting bias assessment	14	Describe any methods used to assess risk of bias due to missing results in a synthesis (arising from reporting biases).	4
Certainty assessment	15	Describe any methods used to assess certainty (or confidence) in the body of evidence for an outcome.	8
**RESULTS**			
Study selection	16a	Describe the results of the search and selection process, from the number of records identified in the search to the number of studies included in the review, ideally using a flow diagram.	4
	16b	Cite studies that might appear to meet the inclusion criteria, but which were excluded, and explain why they were excluded.	4
Study characteristics	17	Cite each included study and present its characteristics.	7 - 8
Risk of bias in studies	18	Present assessments of risk of bias for each included study.	4
Results of individual studies	19	For all outcomes, present, for each study: (a) summary statistics for each group (where appropriate) and (b) an effect estimate and its precision (e.g. confidence/credible interval), ideally using structured tables or plots.	7 – 11, with plots in supplementary material
Results of	20a	For each synthesis, briefly summarise the characteristics and	8

Section and Topic	Item #	Checklist item	Page where item is reported
syntheses		risk of bias among contributing studies.	
	20b	Present results of all statistical syntheses conducted. If meta-analysis was done, present for each the summary estimate and its precision (e.g. confidence/credible interval) and measures of statistical heterogeneity. If comparing groups, describe the direction of the effect.	7 – 11, with plots in supplementary material
	20c	Present results of all investigations of possible causes of heterogeneity among study results.	7 – 11
	20d	Present results of all sensitivity analyses conducted to assess the robustness of the synthesized results.	7 – 11
Reporting biases	21	Present assessments of risk of bias due to missing results (arising from reporting biases) for each synthesis assessed.	4
Certainty of evidence	22	Present assessments of certainty (or confidence) in the body of evidence for each outcome assessed.	7 – 11, with plots in supplementary material
**DISCUSSION**			
Discussion	23a	Provide a general interpretation of the results in the context of other evidence.	12 - 13
	23b	Discuss any limitations of the evidence included in the review.	12 - 13
	23c	Discuss any limitations of the review processes used.	12 - 13
	23d	Discuss implications of the results for practice, policy, and future research.	12 - 14
**OTHER INFORMATION**			
Registration and protocol	24a	Provide registration information for the review, including register name and registration number, or state that the review was not registered.	5
	24b	Indicate where the review protocol can be accessed, or state that a protocol was not prepared.	5
	24c	Describe and explain any amendments to information provided at registration or in the protocol.	
Support	25	Describe sources of financial or non-financial support for the review, and the role of the funders or sponsors in the review.	14
Competing interests	26	Declare any competing interests of review authors.	14
Availability of data, code and other materials	27	Report which of the following are publicly available and where they can be found: template data collection forms; data extracted from included studies; data used for all analyses; analytic code; any other materials used in the review.	4, with table of the data collected in the supporting information

Table S2. Data of the articles included in the review.

Title	Autor	Year	Journal	Mycotoxin	Methods	n	Risk of bias
Aflatoxin and fumonisin contamination of corn (maize, Zea mays) hybrids in Arkansas	ABBAS, et al.	2005	Crop Protection	AFL, FUM	HPLC, ELISA, FLD, MS/MS	59	Low
Aflatoxin contamination of corn under different agro-environmentalconditions and biocontrol applications	ACCINELLI, et al.	2014	Crop Protection	AFL	HPLC, FLD	2	Low
Aflatoxin-free transgenic maize using host-induced gene silencing	THAKARE, et al.	2017	Science Advances	AFL	TLC, UV	5	Low
Aggressiveness and Fumonisins Production of Fusarium Subglutinans and Fusarium Temperatumon Korean Maize Cultivars	TAGELE, et al.	2019	Agronomy	FUM	ELISA	30	Low
Biotic and abiotic factors efficacy of Bt corn in indirectly reducing mycotoxin levels in commercial fields	DOWD	2001	Journal of Economic Entomology	AFL, FUM	HPLC	9	Low
Bt maize can provide non-chemical pest control and enhance food safety in China	YANG, et al.	2022	Plant Biotechnology Journal	FUM	MS/MS	8	Low
Comparative study on concentrations of deoxynivalenol and zearalenone in kernels of transgenic Bt maize hybrids and nontransgenic maize hybrids	VALENTA, et al.	2001	Mycotoxin Research	DON, ZEA	ELISA, HPLC DAD	55	Low
Comparison of fumonisin concentrations in kernels of transgenic Bt maize hybrids and nontransgenic hybrids	MUNKVOLD, et al.	1999	Plant Disease	FUM	FLD	75	Low
Comparison of Fumonisin Contamination Using HPLC and ELISAMethods inBtand Near-Isogenic Maize Hybrids Infested withEuropean Corn Borer or Western Bean Cutworm	BOWERS, et al.	2014	Journal of Agricultural and Food Chemistry	FUM	HPLC FLD, ELISA	227	Low

Comparison of integrated field programmes for the reduction of fumonisin contamination in maize kernels	BLANDINO, et al.	2009	Field Crops Research	FUM	MS/MS	4	Low
Control of Aspergillus flavus growth and aflatoxin production in transgenic maize kernels expressing a tachyplesin-derived synthetic peptide, AGM182	RAJASEKARAN, et al.	2018	Plant Science	AFL	FluoroQuant Afla Test	4	Low
Delaying harvest for naturally drying maize grain increases the risk of kernel rot and fumonisin contamination	COSTA, et al.	2017	Tropical Plant Pathology	FUM	Vicam	6	Low
Determination of Mycotoxin Production ofFusariumSpecies in Genetically Modified MaizeVarieties by Quantitative Flow Immunocytometry	BÁNÁTI, et al.	2016	Toxins	AFL, FUM, DON, ZEA	Flow cytometric analytical assay - Fungi-Plex™ multiplex mycotoxin assay kit	-	Uncertain
Downregulation of transcription factor aflR in Aspergillus flavus confers reduction to aflatoxin accumulation in transgenic maize with alteration of host plant architecture	MASANGA, et al.	2015	Plant Cell Reports	AFL	ELISA	55	Low
Dynamic of water activity in maize hybrids is crucial for fumonisin contaminationin kernels	BATTILANI, et al.	2011	Journal of Cereal Science	FUM	VICAM, HPLC	10	Low
Dynamics of Mycotoxin andAspergillus flavusLevels in Aging Bt and Non-Bt Corn Residues under Mississippi No-Till Conditions	ABBAS, et al.	2008	Journal of Agricultural and Food Chemistry	AFL, FUM, DON, ZEA	HPLC, GC-MS, ELISA, LC/ESSI/MS, MS/MS	3	Low
Effect of Sowing Time on Toxigenic Fungal Infection and Mycotoxin Contamination of Maize Kernels	BLANDINO, et al.	2008	Journal of Phytopathology	AFL, FUM, DON, ZEA	HPLC	2	Low

Efficacy of water-dispersible formulations of biological control strains of Aspergillus flavus for aflatoxin management in corn	WEAVER, et al.	2016	FOOD ADDITIVES & CONTAMINANTS: PART A	AFL	LC, FLD	-	Low
Environmental effects on resistance gene expression in milk stage popcorn kernels and associations with mycotoxin production	DOWD; JHONSON	2015	Mycotoxin Research	AFL, FUM, DON	HPLC	6	Low
FUM Gene Expression Profile and Fumonisin Production by Fusarium verticillioides Inoculated in Bt and Non-Bt Maize	ROCHA, et al.	2015	Frontiers in Microbiology	FUM	LC-10AD	30	Low
Fumonisin in Conventional and Transgenic, Insext-Resistant Maize Intended for Fuel Ethanol Production: Implications for Fermentation Efficiency and DDGS Co-Product Quality	BOWERS; MUNKVOLD	2014	Toxins	FUM	HPLC, FLD	6	Low
FUMONISINS CONCENTRATIONS IN MAIZE AS AFFECTED BY PHYSICO-CHEMICAL, ENVIRONMENTAL AND AGRONOMICAL CONDITIONS	HERRERA, et al.	2010	Maydica	FUM	ROSA	58	Low
Fungal diversity and metabolomic profiles in GM and isogenicnon-GM maize cultivars from Brazil	GASPERINI, et al.	2021	Mycotoxin Research	FUM	LC-MS/MS	36	Low
Fungal Growth andFusariumMycotoxin Content in IsogenicTraditional Maize and Genetically Modified Maize Grown inFrance and Spain	BAKAN, et al.	2002	Journal of Agricultural and Food Chemistry	FUM, DON, ZEA	HPLC FLD, GC-ECD	50	Low
Fusarium and fumonisin in GM maize grown by small-scale farmers in KwaZulu-Natal, South Africa	RHEEDER; WESTHUIZEN	2024	South African Journal of Science	FUM	HPLC	41	Uncertain

Fusarium ear rot and fumonisins in maize kernels when comparing aBthybrid with its non-Btisohybrid and under conventional insecticidecontrol ofBusseola fuscainfestations	NCUBE, et al.	2018	Crop Protection	FUM	HPLC, FLD	6	Low
Fusarium graminearum Mycotoxins in Maize Associated With Striacosta albicosta (Lepidoptera: Noctuidae) Injury	SMITH, et al.	2018	Journal of Economic Entomology	FUM, DON, ZEA	ELISA, LC MS/MS	1000	Low
Fusarium verticillioidesand fumonisin contamination inBtand non-Btmaize cultivated in Brazil	BARROSO, et al.	2017	Mycotoxin Research	FUM	FLD	160	Low
Genetic Variation for Resistance to Ear Rots and Mycotoxins Contamination in Early European Maize Inbred Lines	BOLDUAN, et al.	2009	Crop Science	FUM, DON, ZEA	RIDASCREEN®FAST	21	Low
Hybrid insect protection and fungicide application for managing ear rots and mycotoxins in silage corn	KAUR, et al.	2023	Agronomy Journal	DON, ZEA	MS/MS	-	Low
Impact of transgenic Bt maize residues on the mycotoxigenic plant pathogen Fusarium graminearum and the biocontrol agent Trichoderma atroviride	NAEF, et al.	2006	Journal of Environmental Quality	DON	ELISA	4	Low
Influence of Agricultural Practices on the Contamination ofMaize by Fumonisin Mycotoxins	ARIÑO, et al.	2009	Journal of Food Protection	FUM	ROSA	1	Low
Integration of biological control and transgenic insect protection for mitigation of mycotoxins in corn	WEAVER, et al.	2017	Crop Protection	AFL, FUM	HPLC, FLD	2	Low
Lower Fumonisin Mycotoxin Levels in the Grain of Bt Corn Grown in the United States in 2000–2002	HAMMOND, et al.	2004	Journal of Agricultural and Food Chemistry	AFL, FUM, DON	HPLC, FLD	16	Low

Lower mycotoxin levels in Bt maize grain	FOLCHER, et al.	2010	Agronomy for Sustainable Development	FUM, DON, ZEA	LC-MS/MS, HPLC	84	Low
Modeling effects of environment, insect damage, and Bt genotypes on fumonisin accumulation in maize in Argentina and the Philippines	DE LA CAMPA, et al.	2005	Mycopathologia	FUM	HPLC, FLD	1	Low
PR10 expression in maize and its effect on host resistance against Aspergillus flavus infection and aflatoxin production	CHEN, et al.	2010	MOLECULAR PLANT PATHOLOGY	AFL	-	15	Low
Presence of transgenic proteins and their effect on the content of tannins and aflatoxins in commercial corn	BETANCOURT	2013	Revista Mexicana de Ciencias Agrícolas	AFL	RIDASCREEN®FAST	11	Low
Reduced Contamination by the Fusarium Mycotoxin Zearalenone in Maize Kernels through Genetic Modification with a Detoxification Gene	IGAWA, et al.	2007	APPLIED AND ENVIRONMENTAL MICROBIOLOGY	ZEA	HPLC, FLD	-	Low
Resilience of Biocontrol for Aflatoxin Minimization Strategies: Climate Change Abiotic Factors May Affect Control in Non-GM and GM-Maize Cultivars	GASPERINI, et al.	2019	Frontiers in Microbiology	AFL	HPLC, FLD	-	Uncertain
RNA interference-based silencing of the alpha-amylase (amy1) gene in Aspergillus flavus decreases fungal growth and aflatoxin production in maize kernels	GILBERT, et al.	2018	Planta	AFL	UPLC, FLD	4	Low
Severity of Fusarium ear rot and concentration of fumonisin in grain of Argentinian maize hybrids	PRESELLO, et al.	2007	Crop Protection	FUM	RIDASCREEN®FAST	16	Low

Southwestern corn borer damage and aflatoxin accumulation inconventional and transgenic corn hybrids	WILLIAMS, et al.	2004	Field Crops Research	AFL	VICAM	80	Low
The Pathogenesis-Related Maize Seed (PRms) Gene Plays a Role in Resistance to Aspergillus flavus Infection and Aflatoxin Contamination	MAJUMDAR, et al.	2017	Frontiers in Plant Science	AFL	UPLC, FLD	-	Low
Transgenic versus conventional corn: fate of fumonisins during industrial dry milling	BORDINI, et al.	2019	Mycotoxin Research	FUM	HPLC	480	Low

Figure S1. Forest plot of the overall analysis of mycotoxin quantity, with all experiments included.

Study or Subgroup	Convencional Mean	SD	Total	Transgenic Mean	SD	Total	Weight	Mean Difference IV, Fixed, 95% CI
ABBAS 2005 AFL 99	0.215	0.049	17	0.197	0.085	4	0.0%	0.02 [-0.07, 0.10]
ABBAS 2005 AFL E98	0.227	0.046	17	0.196	0.086	4	0.0%	0.03 [-0.06, 0.12]
ABBAS 2005 AFL H98	0.0266	0.0175	15	0.0036	0.0018	14	0.1%	0.02 [0.01, 0.03]
ABBAS 2005 FUM 01	48.1	4.2	17	38.2	6.1	4	0.0%	9.90 [3.60, 16.20]
ABBAS 2005 FUM 99	45.6	6	17	39.6	4.2	4	0.0%	6.00 [0.99, 11.01]
ABBAS 2005 FUM E98	3.32	0.6	15	2.09	0.25	14	0.0%	1.23 [0.90, 1.56]
ABBAS 2005 FUM H98	36.35	3.45	14	42.35	6.25	4	0.0%	-6.00 [-12.39, 0.39]
ABBAS 2008 AFL	0.211	0.0259	5	0.109	0.0143	5	0.0%	0.10 [0.08, 0.13]
ACCINELLI 2014 AFL	0.0562	0.0056	1	0.1354	0.008	1	0.0%	-0.08 [-0.10, -0.06]
BORDINI 2019 FUM	1.17	0.62	240	0.99	0.3	240	0.0%	0.18 [0.09, 0.27]
BOWERS 2014 FUM 08	6.6	8.62	2	1.52	2.03	4	0.0%	5.08 [-7.03, 17.19]
BOWERS 2014 FUM 09	0.67	1.08	3	0.28	0.47	6	0.0%	0.39 [-0.89, 1.67]
BOWERS 2014 FUM 10	0.76	0.93	3	0.47	0.59	6	0.0%	0.29 [-0.86, 1.44]
DOWD 2001 FUM 98	1.08	0.27	2	0.43	0.13	4	0.0%	0.65 [0.25, 1.05]
DOWD 2001 FUM 991	0.5	0.19	1	0.54	0.27	5	0.0%	-0.04 [-0.48, 0.40]
FOLCHER 2010 DON 05	0.113576	0.01248	21	0.18569	0.0102	21	0.1%	-0.07 [-0.08, -0.07]
FOLCHER 2010 DON 06	0.238805	0.01224	21	0.97561	0.10295	21	0.0%	-0.74 [-0.78, -0.69]
FOLCHER 2010 FUM 05	6.12	0.28208	21	0.26562	0.02489	21	0.0%	5.85 [5.73, 5.98]
FOLCHER 2010 FUM 06	5.62	0.31717	21	0.42508	0.05437	21	0.0%	5.19 [5.06, 5.33]
FOLCHER 2010 ZEA 05	0.01895	0.00193	21	0.009373	0.00066	21	7.7%	0.01 [0.01, 0.01]
FOLCHER 2010 ZEA 06	0.00347	0.0005	21	0.001567	0.00031	21	92.0%	0.00 [0.00, 0.00]
HERRERA 2010 FUM	0.673	0.02557	49	0.405	0.04	9	0.0%	0.27 [0.24, 0.30]
NAEF 2006 DON 02	1.79	0.11	24	1.25	0.11	24	0.0%	0.54 [0.48, 0.60]
NAEF 2006 DON 03	0.52	0.035	24	0.52	0.033	24	0.0%	0.00 [-0.02, 0.02]
RHEEDER 2024 FUM	0.897	1.38	155	0.538	0.918	183	0.0%	0.36 [0.10, 0.61]
ROCHA 2016 FUM T11	5.93	0.039	80	4.48	0.05	80	0.0%	1.45 [1.44, 1.46]
ROCHA 2016 FUM T21	3.19	0.038	80	2.76	0.037	80	0.0%	0.43 [0.42, 0.44]
WEAVER 2017 AFL 1	0.8	0.071	6	0.8	0.071	6	0.0%	0.00 [-0.08, 0.08]
WEAVER 2017 FUM 1	0.043	0.011	6	0.053	0.013	6	0.0%	-0.01 [-0.02, 0.00]
YANG 2022 FUM T1 19	6.68	15.84	8	0.56	0.85	8	0.0%	6.12 [-4.87, 17.11]
YANG 2022 FUM T1 20	5.12	6.75	9	0.74	1.59	9	0.0%	4.38 [-0.15, 8.91]
YANG 2022 FUM T2 19	15.68	32.13	9	1.69	3.69	9	0.0%	13.99 [-7.14, 35.12]
YANG 2022 FUM T2 20	5.59	6.87	9	0.25	0.3	9	0.0%	5.34 [0.85, 9.83]
**Total (95% CI)**			**954**			**892**	**100.0%**	**0.00 [0.00, 0.00]**

Heterogeneity: $Chi^2 = 64014.04$, $df = 32$ ($P < 0.00001$); $I^2 = 100\%$
Test for overall effect: $Z = 25.00$ ($P < 0.00001$)

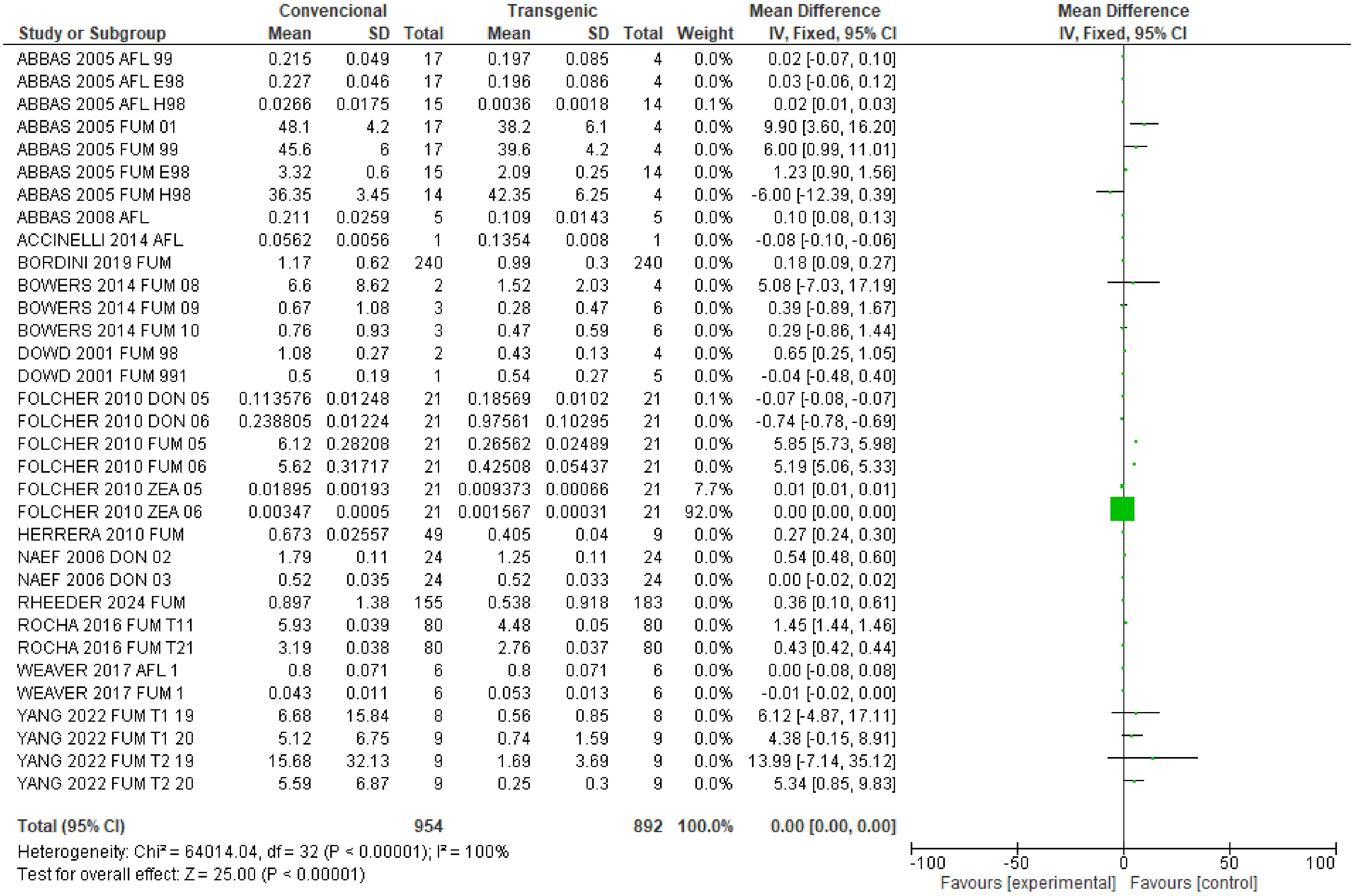

Figure S2. Funnel Plot of the overall analysis of mycotoxin quantity, with all experiments included.

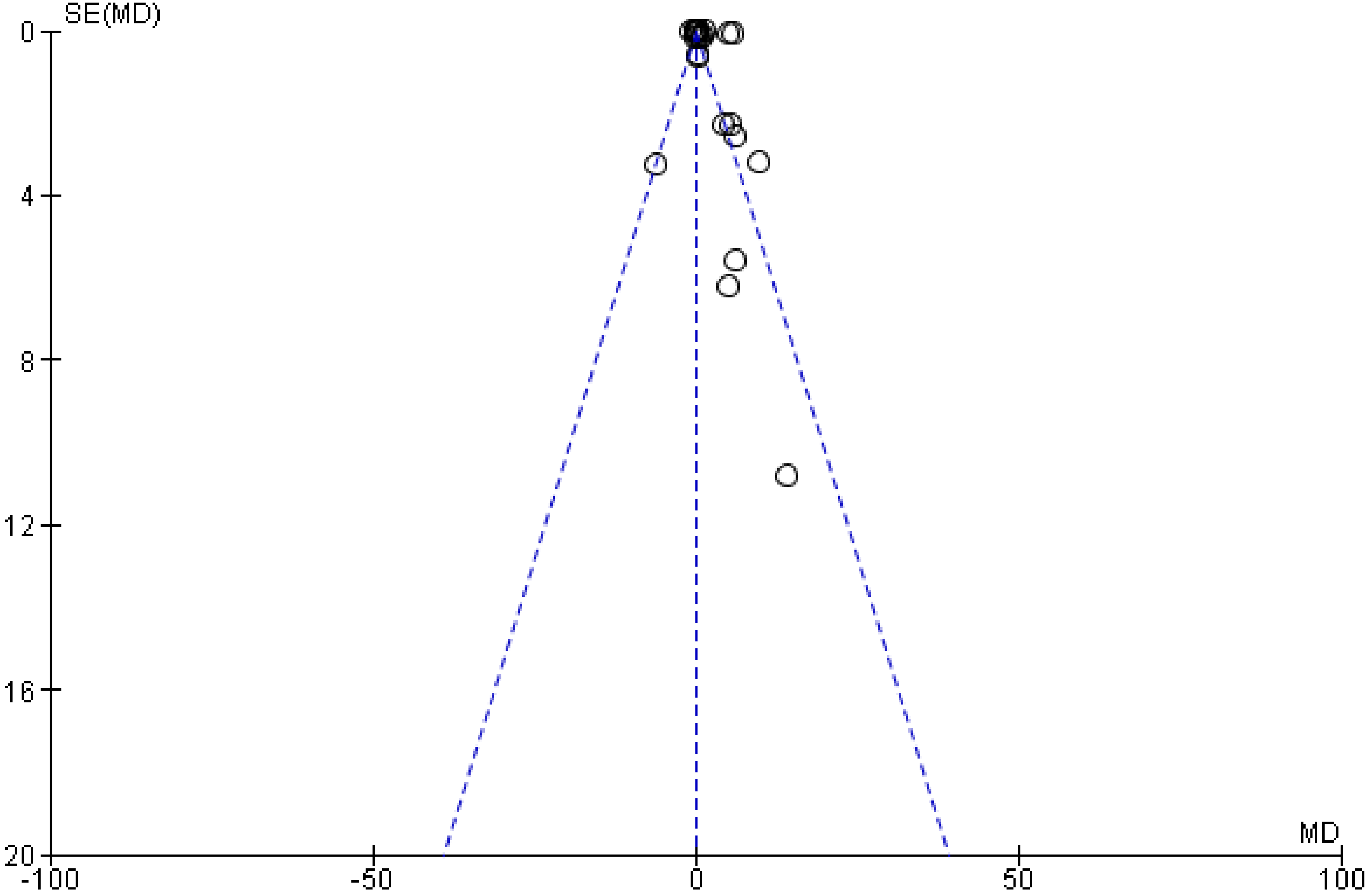

SE(MD)
0
4
8
12
16
20
MD
-100
-50
0
50
100

Figure S3. Forest Plot of the overall analysis of mycotoxin quantity, without the three experiments with discrepant mycotoxin amounts.

Study or Subgroup	Convencional Mean	SD	Total	Transgenic Mean	SD	Total	Weight	Mean Difference IV, Fixed, 95% CI
ABBAS 2005 AFL 99	0.215	0.049	17	0.197	0.085	4	0.0%	0.02 [-0.07, 0.10]
ABBAS 2005 AFL E98	0.227	0.046	17	0.196	0.086	4	0.0%	0.03 [-0.06, 0.12]
ABBAS 2005 AFL H98	0.0266	0.0175	15	0.0036	0.0018	14	0.1%	0.02 [0.01, 0.03]
ABBAS 2005 FUM 99	3.32	0.6	15	2.09	0.25	14	0.0%	1.23 [0.90, 1.56]
ABBAS 2008 AFL	0.211	0.0259	5	0.109	0.0143	5	0.0%	0.10 [0.08, 0.13]
ACCINELLI 2014 AFL	0.0562	0.0056	1	0.1354	0.008	1	0.0%	-0.08 [-0.10, -0.06]
BORDINI 2019 FUM	1.17	0.62	240	0.99	0.3	240	0.0%	0.18 [0.09, 0.27]
BOWERS 2014 FUM 08	6.6	8.62	2	1.52	2.03	4	0.0%	5.08 [-7.03, 17.19]
BOWERS 2014 FUM 09	0.67	1.08	3	0.28	0.47	6	0.0%	0.39 [-0.89, 1.67]
BOWERS 2014 FUM 10	0.76	0.93	3	0.47	0.59	6	0.0%	0.29 [-0.86, 1.44]
DOWD 2001 FUM 98	1.08	0.27	2	0.43	0.13	4	0.0%	0.65 [0.25, 1.05]
DOWD 2001 FUM 991	0.5	0.19	1	0.54	0.27	5	0.0%	-0.04 [-0.48, 0.40]
FOLCHER 2010 DON 05	0.113576	0.01248	21	0.18569	0.0102	21	0.1%	-0.07 [-0.08, -0.07]
FOLCHER 2010 DON 06	0.238805	0.01224	21	0.97561	0.10295	21	0.0%	-0.74 [-0.78, -0.69]
FOLCHER 2010 FUM 05	6.12	0.28208	21	0.26562	0.02489	21	0.0%	5.85 [5.73, 5.98]
FOLCHER 2010 FUM 06	5.62	0.31717	21	0.42508	0.05437	21	0.0%	5.19 [5.06, 5.33]
FOLCHER 2010 ZEA 05	0.01895	0.00193	21	0.009373	0.00066	21	7.7%	0.01 [0.01, 0.01]
FOLCHER 2010 ZEA 06	0.00347	0.0005	21	0.001567	0.00031	21	92.0%	0.00 [0.00, 0.00]
HERRERA 2010 FUM	0.673	0.02557	49	0.405	0.04	9	0.0%	0.27 [0.24, 0.30]
NAEF 2006 DON 02	1.79	0.11	24	1.25	0.11	24	0.0%	0.54 [0.48, 0.60]
NAEF 2006 DON 03	0.52	0.035	24	0.52	0.033	24	0.0%	0.00 [-0.02, 0.02]
RHEEDER 2024 FUM	0.897	1.38	155	0.538	0.918	183	0.0%	0.36 [0.10, 0.61]
ROCHA 2016 FUM T11	5.93	0.039	80	4.48	0.05	80	0.0%	1.45 [1.44, 1.46]
ROCHA 2016 FUM T21	3.19	0.038	80	2.76	0.037	80	0.0%	0.43 [0.42, 0.44]
WEAVER 2017 AFL 1	0.8	0.071	6	0.8	0.071	6	0.0%	0.00 [-0.08, 0.08]
WEAVER 2017 FUM 1	0.043	0.011	6	0.053	0.013	6	0.0%	-0.01 [-0.02, 0.00]
YANG 2022 FUM T1 19	6.68	15.84	8	0.56	0.85	8	0.0%	6.12 [-4.87, 17.11]
YANG 2022 FUM T1 20	5.12	6.75	9	0.74	1.59	9	0.0%	4.38 [-0.15, 8.91]
YANG 2022 FUM T2 19	15.68	32.13	9	1.69	3.69	9	0.0%	13.99 [-7.14, 35.12]
YANG 2022 FUM T2 20	5.59	6.87	9	0.25	0.3	9	0.0%	5.34 [0.85, 9.83]
**Total (95% CI)**			**906**			**880**	**100.0%**	**0.00 [0.00, 0.00]**

Heterogeneity: Chi² = 63995.66, df = 29 (P < 0.00001); I² = 100%
Test for overall effect: Z = 25.00 (P < 0.00001)

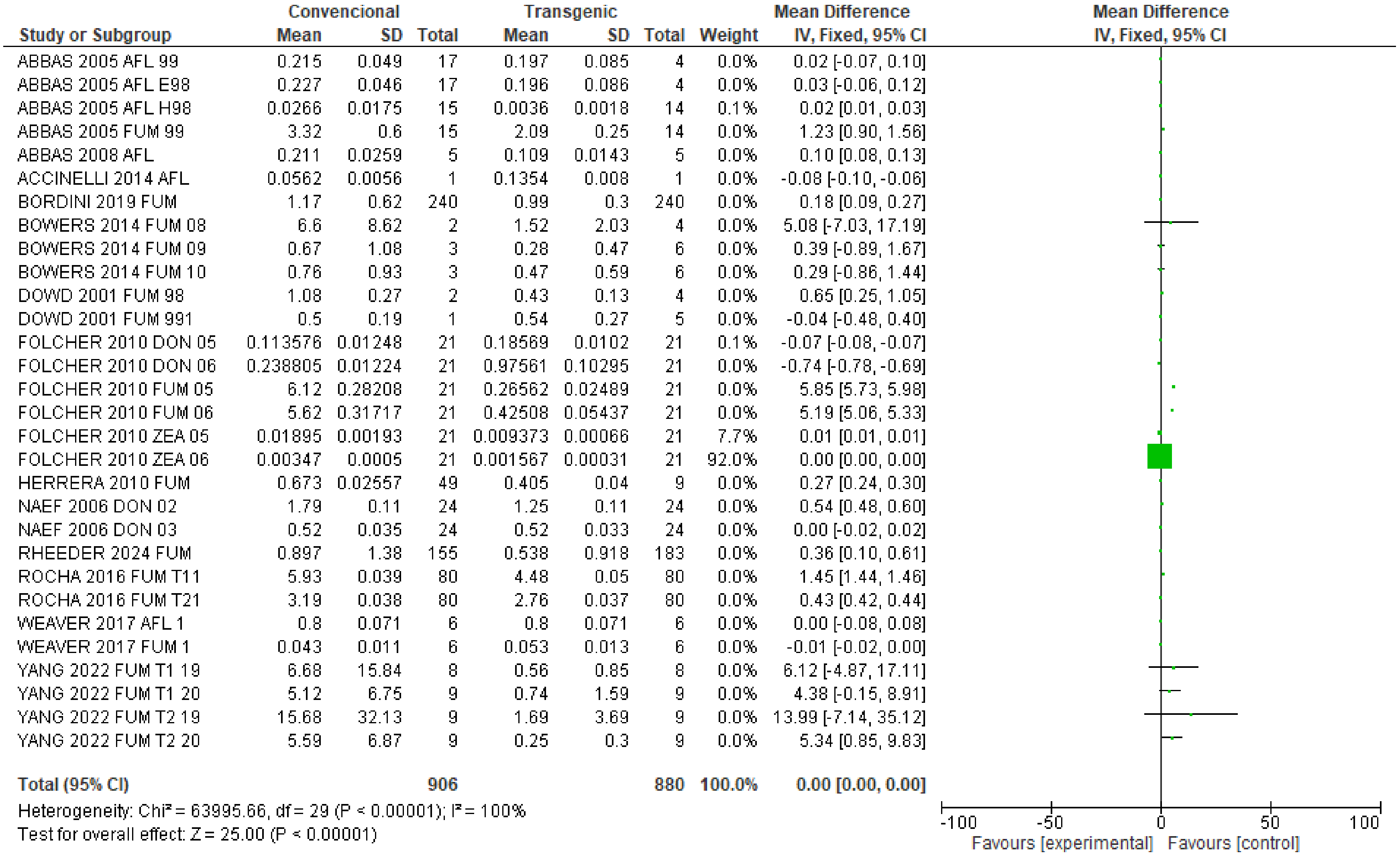

Figure S4. Funnel Plot of the overall analysis of mycotoxin quantity, without the three experiments with discrepant mycotoxin amounts.

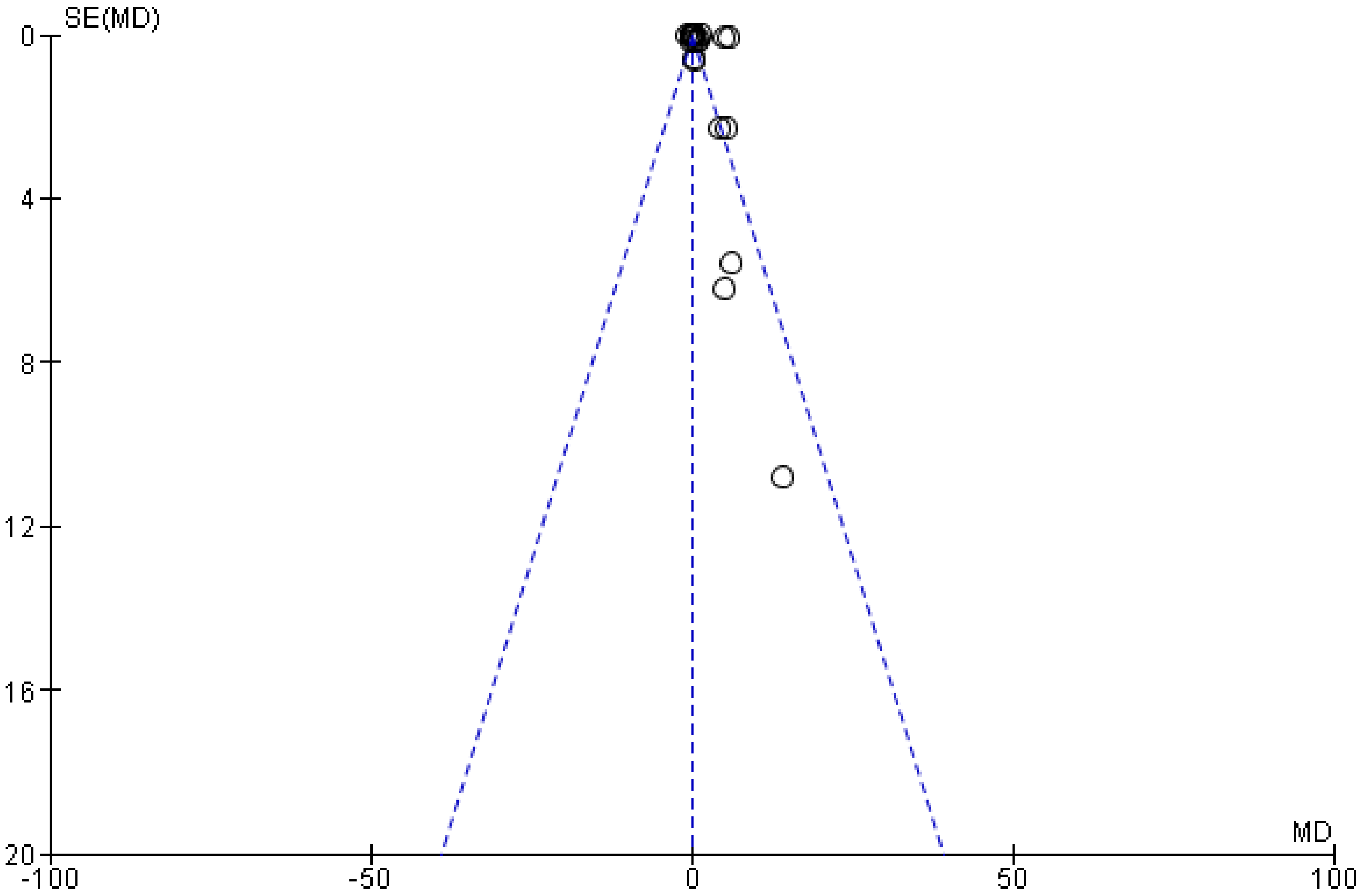
SE(MD)
0
4
8
12
16
20
MD
-100
-50
0
50
100

Figure S5. Forest plot of the fumonisin analysis of mycotoxin quantity, with all experiments included.

	Convencional			Transgenic				Mean Difference
Study or Subgroup	Mean	SD	Total	Mean	SD	Total	Weight	IV, Fixed, 95% CI
ABBAS 2005 FUM 01	48.1	4.2	17	38.2	6.1	4	0.0%	9.90 [3.60, 16.20]
ABBAS 2005 FUM 99	45.6	6	17	39.6	4.2	4	0.0%	6.00 [0.99, 11.01]
ABBAS 2005 FUM E98	3.32	0.6	15	2.09	0.25	14	0.1%	1.23 [0.90, 1.56]
ABBAS 2005 FUM H98	36.35	3.45	14	42.35	6.25	4	0.0%	-6.00 [-12.39, 0.39]
BORDINI 2019 FUM	1.17	0.62	240	0.99	0.3	240	0.9%	0.18 [0.09, 0.27]
BOWERS 2014 FUM 08	6.6	8.62	2	1.52	2.03	4	0.0%	5.08 [-7.03, 17.19]
BOWERS 2014 FUM 09	0.67	1.08	3	0.28	0.47	6	0.0%	0.39 [-0.89, 1.67]
BOWERS 2014 FUM 10	0.76	0.93	3	0.47	0.59	6	0.0%	0.29 [-0.86, 1.44]
DOWD 2001 FUM 98	1.08	0.27	2	0.43	0.13	4	0.0%	0.65 [0.25, 1.05]
DOWD 2001 FUM 991	0.5	0.19	1	0.54	0.27	5	0.0%	-0.04 [-0.48, 0.40]
FOLCHER 2010 FUM 05	6.12	0.28208	21	0.26562	0.02489	21	0.5%	5.85 [5.73, 5.98]
FOLCHER 2010 FUM 06	5.62	0.31717	21	0.42508	0.05437	21	0.4%	5.19 [5.06, 5.33]
HERRERA 2010 FUM	0.673	0.02557	49	0.405	0.04	9	9.5%	0.27 [0.24, 0.30]
RHEEDER 2024 FUM	0.897	1.38	155	0.538	0.918	183	0.1%	0.36 [0.10, 0.61]
ROCHA 2016 FUM T11	5.93	0.039	80	4.48	0.05	80	36.0%	1.45 [1.44, 1.46]
ROCHA 2016 FUM T21	3.19	0.038	80	2.76	0.037	80	51.4%	0.43 [0.42, 0.44]
WEAVER 2017 FUM 1	0.8	0.071	6	0.8	0.071	6	1.1%	0.00 [-0.08, 0.08]
YANG 2022 FUM T1 19	6.68	15.84	8	0.56	0.85	8	0.0%	6.12 [-4.87, 17.11]
YANG 2022 FUM T1 20	5.12	6.75	9	0.74	1.59	9	0.0%	4.38 [-0.15, 8.91]
YANG 2022 FUM T2 19	15.68	32.13	9	1.69	3.69	9	0.0%	13.99 [-7.14, 35.12]
YANG 2022 FUM T2 20	5.59	6.87	9	0.25	0.3	9	0.0%	5.34 [0.85, 9.83]
**Total (95% CI)**			**761**			**726**	**100.0%**	**0.82 [0.81, 0.83]**

Heterogeneity: Chi² = 25003.23, df = 20 (P < 0.00001); I² = 100%
Test for overall effect: Z = 192.43 (P < 0.00001)

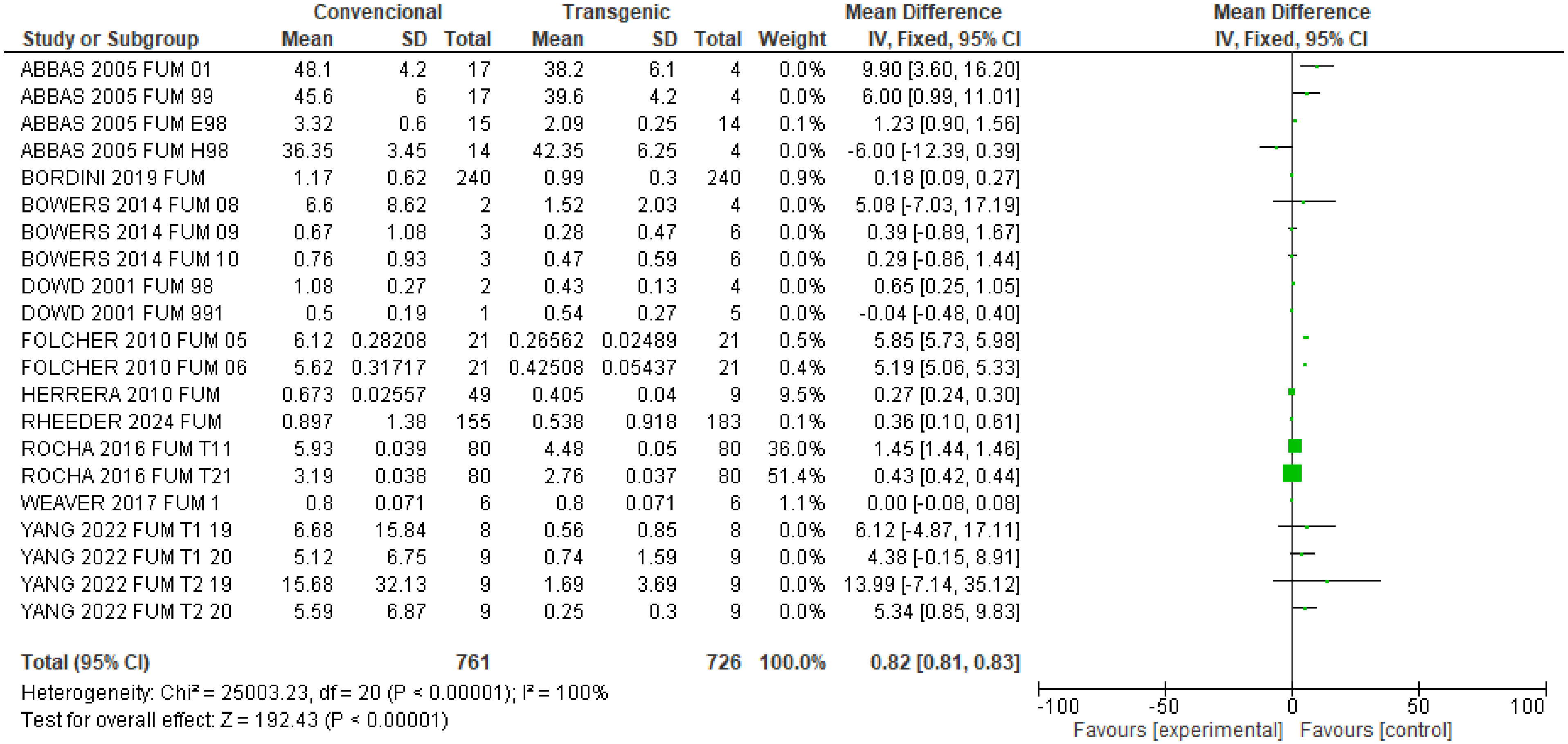

Figure S6. Funnel Plot of the fumonisin analysis of mycotoxin quantity, with all experiments included.

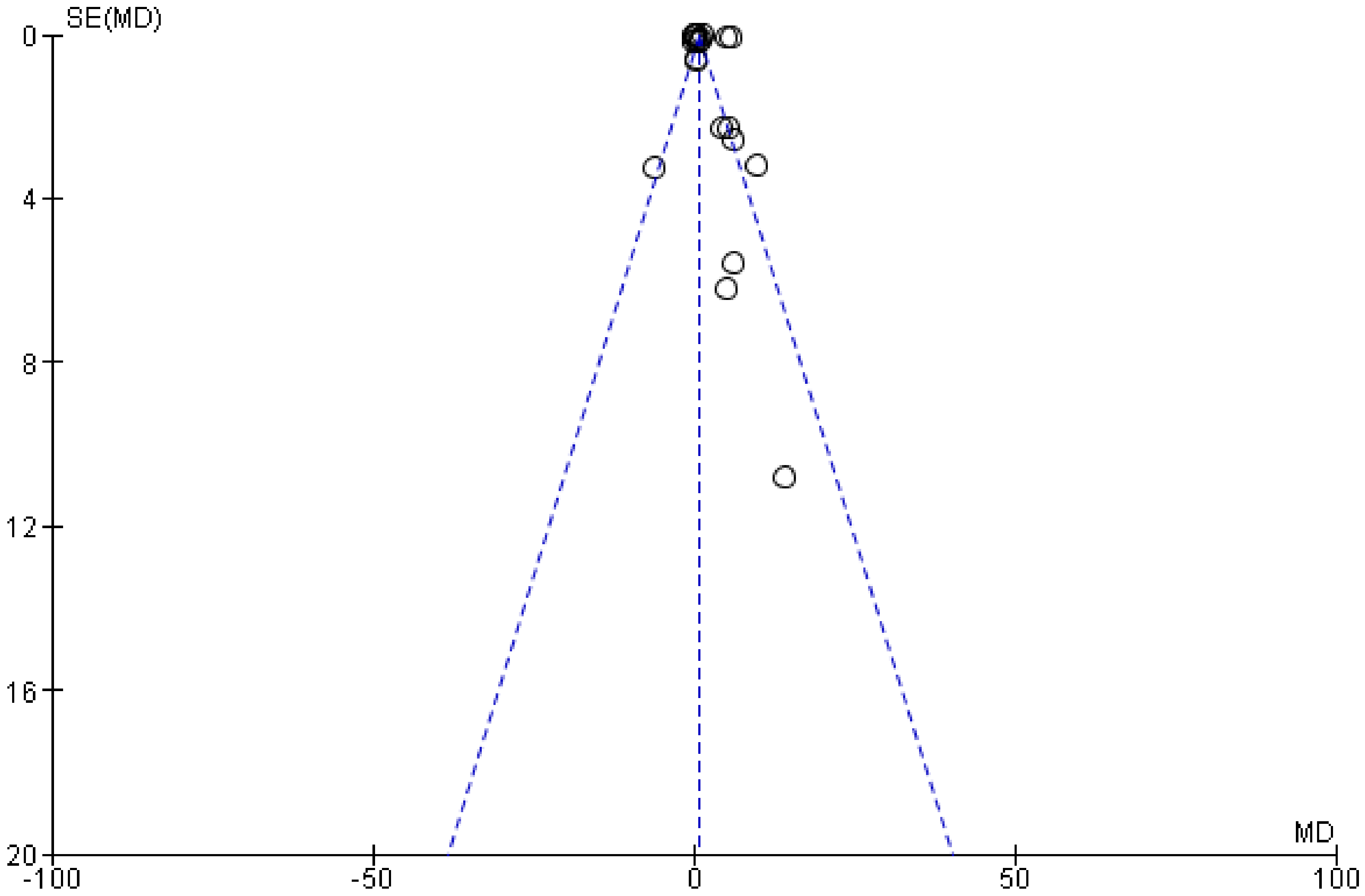

Figure S7. Forest plot of the fumonisin analysis of mycotoxin quantity, without the three experiments with discrepant mycotoxin amounts.

	Convencional			Transgenic				Mean Difference
Study or Subgroup	Mean	SD	Total	Mean	SD	Total	Weight	IV, Fixed, 95% CI
ABBAS 2005 FUM 99	3.32	0.6	15	2.09	0.25	14	0.1%	1.23 [0.90, 1.56]
BORDINI 2019 FUM	1.17	0.62	240	0.99	0.3	240	0.9%	0.18 [0.09, 0.27]
BOWERS 2014 FUM 08	6.6	8.62	2	1.52	2.03	4	0.0%	5.08 [-7.03, 17.19]
BOWERS 2014 FUM 09	0.67	1.08	3	0.28	0.47	6	0.0%	0.39 [-0.89, 1.67]
BOWERS 2014 FUM 10	0.76	0.93	3	0.47	0.59	6	0.0%	0.29 [-0.86, 1.44]
DOWD 2001 FUM 98	1.08	0.27	2	0.43	0.13	4	0.0%	0.65 [0.25, 1.05]
DOWD 2001 FUM 991	0.5	0.19	1	0.54	0.27	5	0.0%	-0.04 [-0.48, 0.40]
FOLCHER 2010 FUM 05	6.12	0.28208	21	0.26562	0.02489	21	0.5%	5.85 [5.73, 5.98]
FOLCHER 2010 FUM 06	5.62	0.31717	21	0.42508	0.05437	21	0.4%	5.19 [5.06, 5.33]
HERRERA 2010 FUM	0.673	0.02557	49	0.405	0.04	9	9.5%	0.27 [0.24, 0.30]
RHEEDER 2024 FUM	0.897	1.38	155	0.538	0.918	183	0.1%	0.36 [0.10, 0.61]
ROCHA 2016 FUM T11	5.93	0.039	80	4.48	0.05	80	36.0%	1.45 [1.44, 1.46]
ROCHA 2016 FUM T21	3.19	0.038	80	2.76	0.037	80	51.4%	0.43 [0.42, 0.44]
WEAVER 2017 FUM 1	0.8	0.071	6	0.8	0.071	6	1.1%	0.00 [-0.08, 0.08]
YANG 2022 FUM T1 19	6.68	15.84	8	0.56	0.85	8	0.0%	6.12 [-4.87, 17.11]
YANG 2022 FUM T1 20	5.12	6.75	9	0.74	1.59	9	0.0%	4.38 [-0.15, 8.91]
YANG 2022 FUM T2 19	15.68	32.13	9	1.69	3.69	9	0.0%	13.99 [-7.14, 35.12]
YANG 2022 FUM T2 20	5.59	6.87	9	0.25	0.3	9	0.0%	5.34 [0.85, 9.83]
**Total (95% CI)**			**713**			**714**	**100.0%**	**0.82 [0.81, 0.83]**

Heterogeneity: Chi² = 24986.76, df = 17 (P < 0.00001); I² = 100%

Test for overall effect: Z = 192.42 (P < 0.00001)

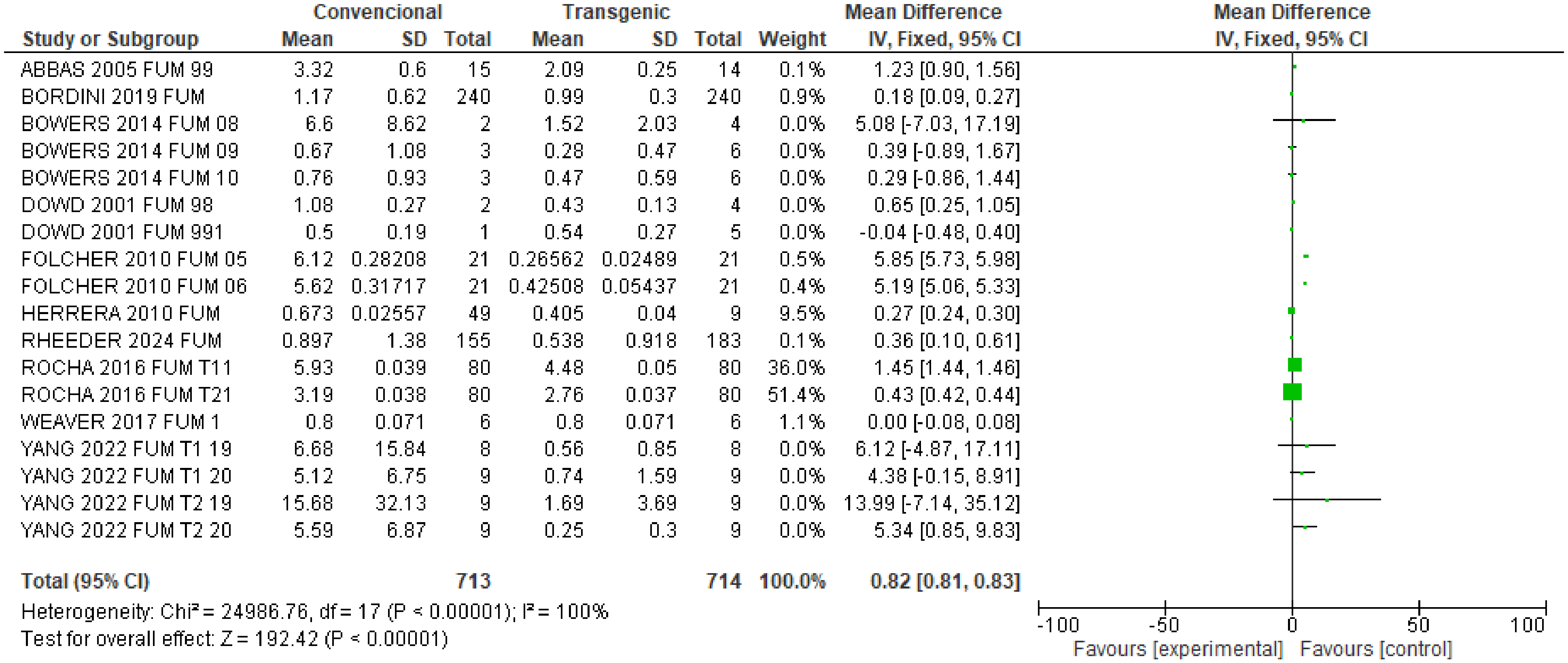

Figure S8. Funnel Plot of the fumonisin analysis of mycotoxin quantity, without the three experiments with discrepant mycotoxin amounts.

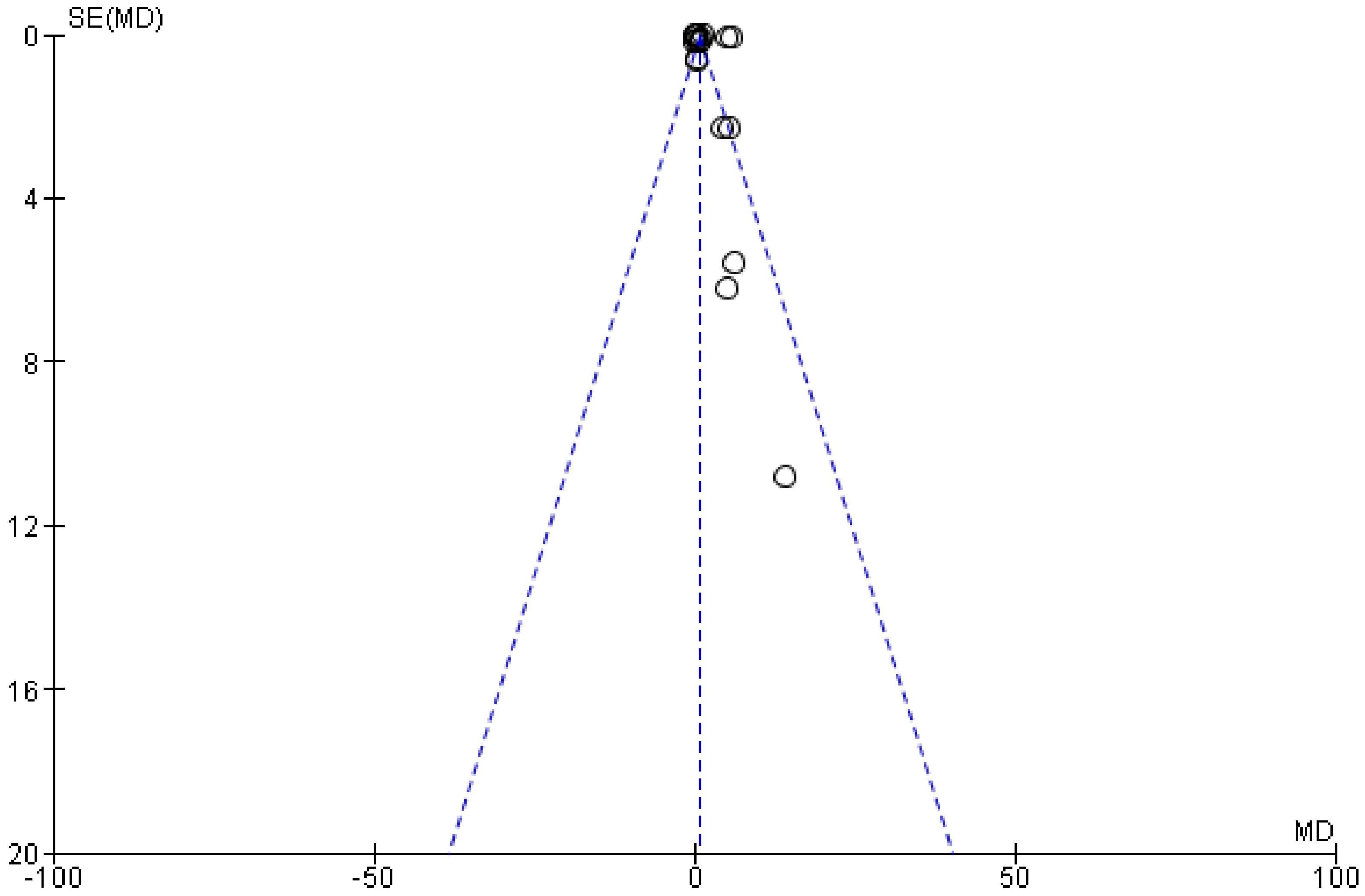

Figure S9. Forest plot of the aflatoxin analysis of mycotoxin quantity.

	Experimental			Control				Mean Difference
**Study or Subgroup**	**Mean**	**SD**	**Total**	**Mean**	**SD**	**Total**	**Weight**	**IV, Fixed, 95% CI**
ABBAS 2005 AFL 99	0.215	0.049	17	0.197	0.085	4	0.6%	0.02 [-0.07, 0.10]
ABBAS 2005 AFL E98	0.227	0.046	17	0.196	0.086	4	0.6%	0.03 [-0.06, 0.12]
ABBAS 2005 AFL H98	0.0266	0.0175	15	0.0036	0.0018	14	56.1%	0.02 [0.01, 0.03]
ABBAS 2008 AFL	0.0562	0.0056	1	0.1354	0.008	1	12.1%	-0.08 [-0.10, -0.06]
ACCINELLI 2014 AFL	0.211	0.0259	5	0.109	0.0143	5	6.6%	0.10 [0.08, 0.13]
WEAVER 2017 AFL	0.043	0.011	6	0.053	0.013	6	24.0%	-0.01 [-0.02, 0.00]
**Total (95% CI)**			**61**			**34**	**100.0%**	**0.01 [0.00, 0.01]**

Heterogeneity: Chi² = 148.13, df = 5 (P < 0.00001); I² = 97%
Test for overall effect: Z = 2.33 (P = 0.02)

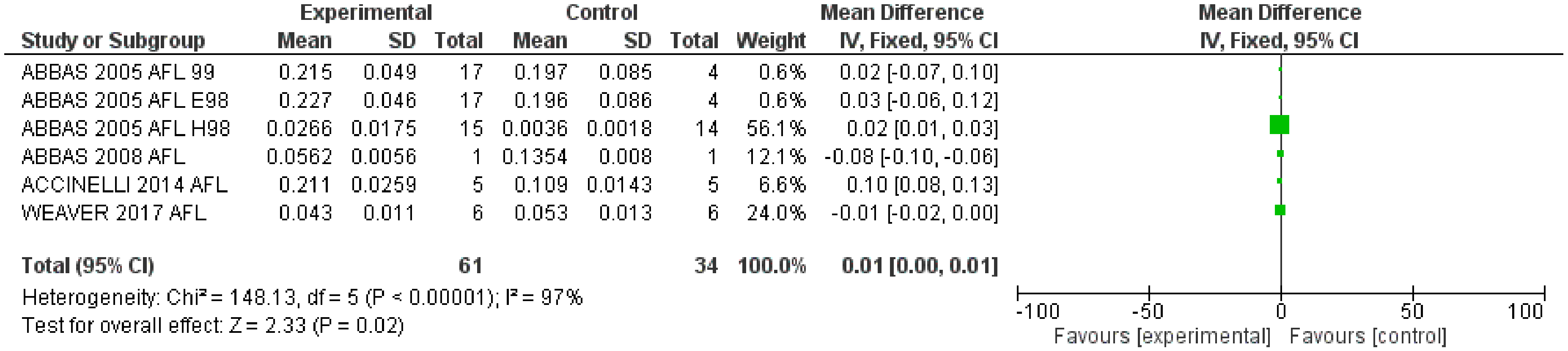

Figure S10. Funnel plot of the aflatoxin analysis of mycotoxin quantity.

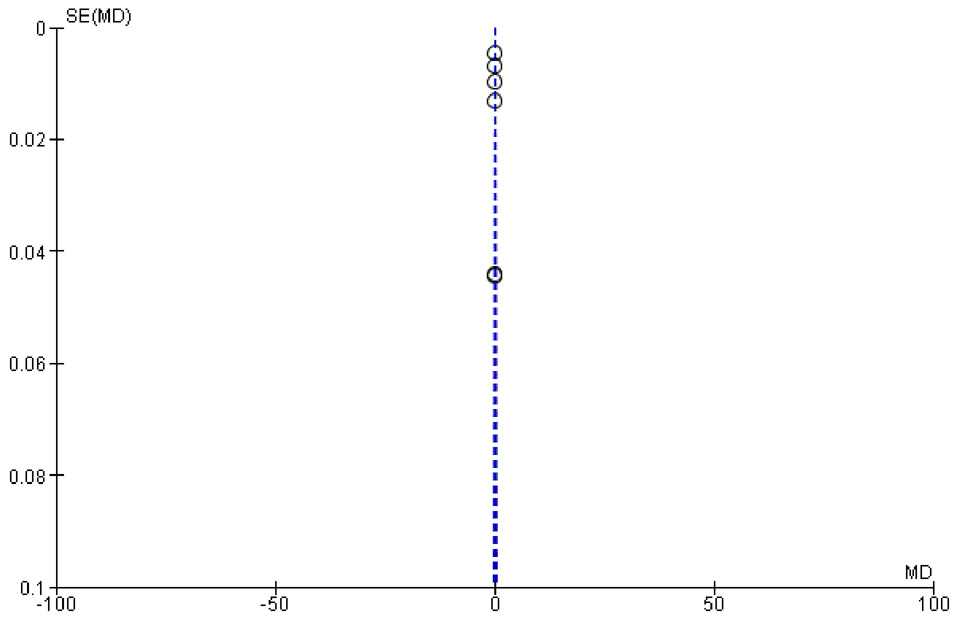

Figure S11. Forest plot of deoxynivalenol analysis of mycotoxin quantity.

	Convencional			Transgênico				Mean Difference
Study or Subgroup	Mean	SD	Total	Mean	SD	Total	Weight	IV, Fixed, 95% CI
FOLCHER 2010 DON 05	0.52	0.035	24	0.52	0.033	24	11.0%	0.00 [-0.02, 0.02]
FOLCHER 2010 DON 06	1.79	0.11	24	1.25	0.11	24	1.1%	0.54 [0.48, 0.60]
NAEF 2006 DON 02	0.238805	0.01224	21	0.97561	0.10295	21	2.1%	-0.74 [-0.78, -0.69]
NAEF 2006 DON 03	0.113576	0.01248	21	0.18569	0.0102	21	85.9%	-0.07 [-0.08, -0.07]
**Total (95% CI)**			**90**			**90**	**100.0%**	**-0.07 [-0.08, -0.07]**

Heterogeneity: $Chi^2 = 1288.69$, $df = 3$ ($P < 0.00001$); $I^2 = 100\%$
Test for overall effect: $Z = 21.94$ ($P < 0.00001$)

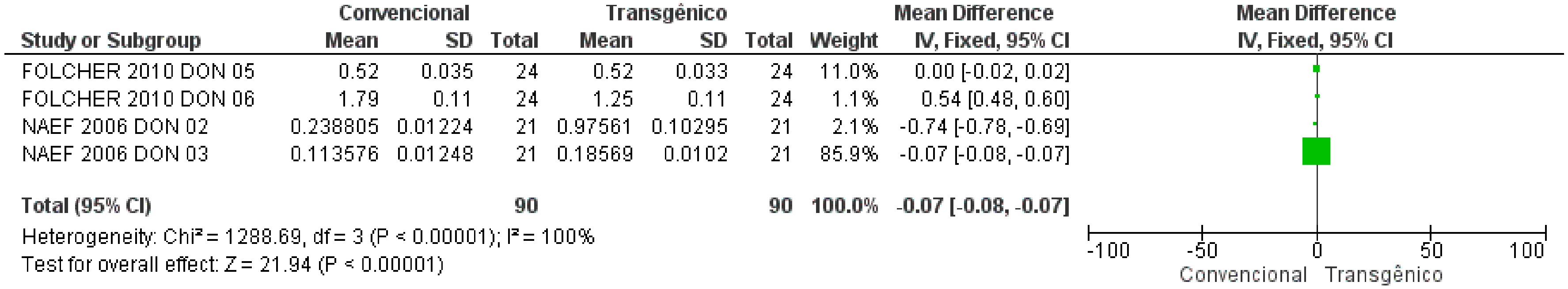

Figure S12. Funnel plot of the deoxynivalenol analysis of mycotoxin quantity.

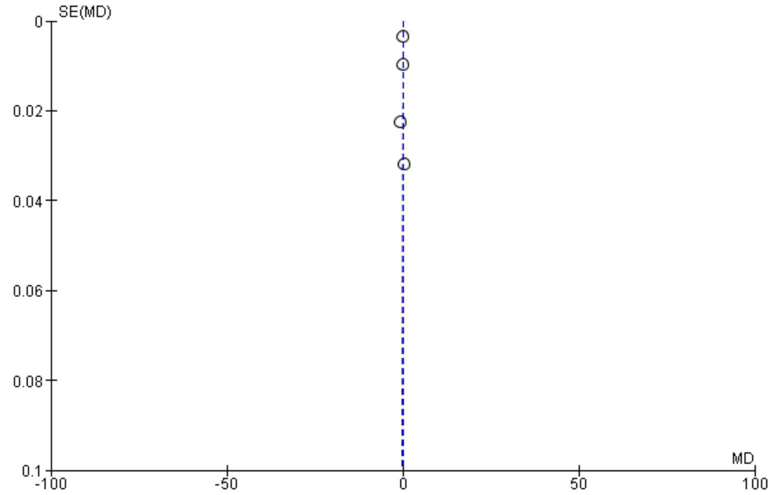

Figure S13. Forest plot of the zearalenone analysis of mycotoxin quantity.

Study or Subgroup	Convencional Mean	Convencional SD	Convencional Total	Trangênico Mean	Trangênico SD	Trangênico Total	Weight	Mean Difference IV, Fixed, 95% CI
FOLCHER 2010 ZEA 05	0.01895	0.00193	21	0.009373	0.00066	21	7.7%	0.01 [0.01, 0.01]
FOLCHER 2010 ZEA 06	0.00347	0.0005	21	0.001567	0.00031	21	92.3%	0.00 [0.00, 0.00]
**Total (95% CI)**			**42**			**42**	**100.0%**	**0.00 [0.00, 0.00]**

Heterogeneity: Chi² = 274.42, df = 1 (P < 0.00001); I² = 100%
Test for overall effect: Z = 20.21 (P < 0.00001)

Mean Difference IV, Fixed, 95% CI

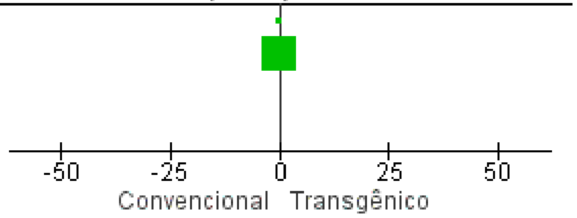

Figure S14. Funnel plot of the zearalenone analysis of mycotoxin quantity.

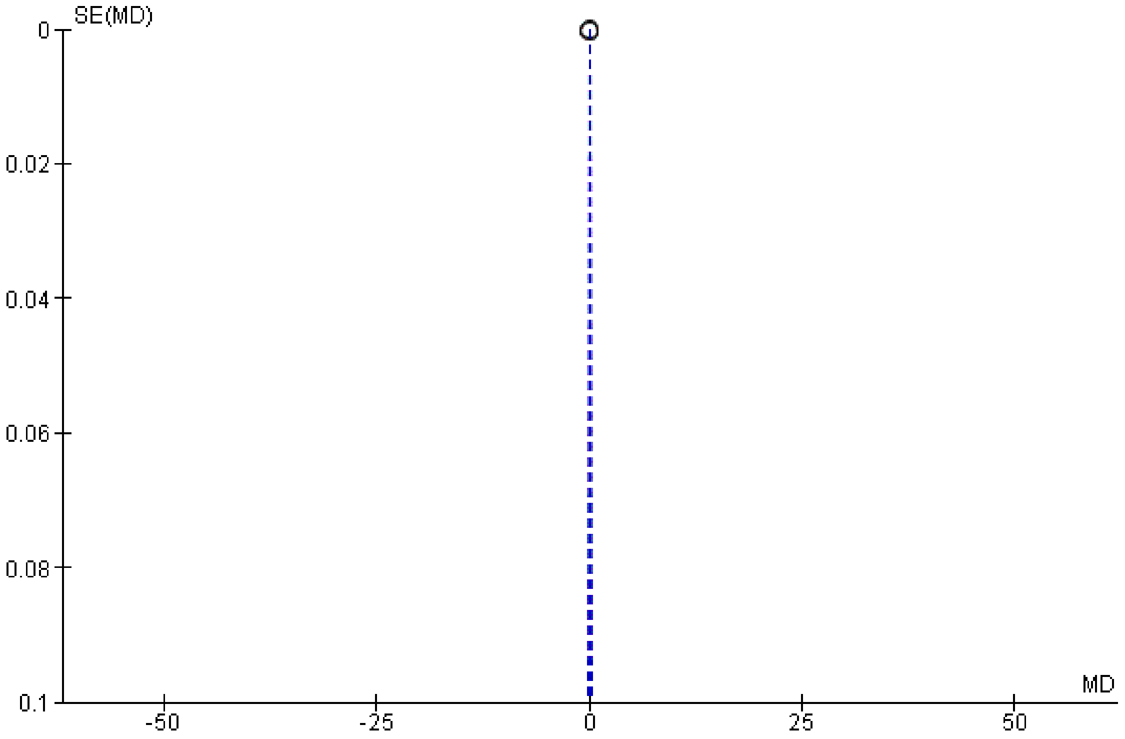

Supplement: Supplementary file 1 [file toxins-16-00373-s001.zip › toxins-3101120-supplementary.pdf]
